# Mentoring Multi-College Bystander Efficacy Evaluation – an Approach to Growing the Next Generation of Gender-Based Interpersonal Violence Intervention and Prevention (VIP) Researchers

**DOI:** 10.1007/s10896-020-00133-9

**Published:** 2020-01-22

**Authors:** Ann L. Coker, Danielle Davidov, Heather M. Bush, Emily R. Clear

**Affiliations:** 1 College of Medicine, University of Kentucky, 800 Rose St., Pavilion H, Room C361, Lexington, KY 40536 USA; 2 College of Public Health, West Virginia University, Morgantown, WV USA; 3 College of Public Health, University of Kentucky, Lexington, KY USA

**Keywords:** Research, Mentorship, Productivity, Collaboration, Violence prevention

## Abstract

The Centers for Disease Control and Prevention provided funding (U01 CE002668) to evaluate bystander program efficacy to reduce gender-based violence on college campuses (Aim 1) and to create a mentoring network (Aim 2) for young campus-based researchers interested in violence intervention or prevention (VIP). While an evaluation of this mentoring program is ongoing, our purpose here was to document the strategies used to create, implement, and begin evaluation of this national multi-college mentoring network. As each public college was recruited into this evaluation named multi-college Bystander Efficacy Evaluation (mcBEE), each college was invited to nominate a researcher interested in receiving mentorship as a mcBEE fellow. Senior faculty with active VIP research careers were recruited as mentors. Mentorship occurred through annual meetings over time (2015–2019), weekly to bimonthly calls or video conferencing with 2–3 other fellows, and a mentor forming a group with 3–4 mentees, termed a hive. The initial focus of hive meetings was 1) creating and maintaining an active daily writing practice and 2) developing productivity plans, to include research, personal, and professional goals. Manuscript and grant writing feedback was provided throughout the network electronically or ‘live’ workshops. Annual surveys were implemented to investigate program efficacy. Our mcBEE team was able to successfully assemble a national network of VIP fellows and provide small group and individualized mentoring. Our ultimate goal was that of supporting our fellows’ own trajectories in gender-based VIP research, teaching, administration, or service. Evaluation of our fellow and mentor cohort is ongoing (2015–2019).

## Introduction

Justification for Mentoring mcBEE. This purpose of this manuscript is to describe a mentoring program developed to support trained researchers seeking a career in gender-based violence research. Our rationale for developing this national mentorship network was based on the unique needs of researchers in this emerging field. Because this field is relatively nascent (Dahlberg and Mercy 2009), there are challenges resulting from having few resources to support those interested in creating a career in the field of gender-based violence intervention and/or prevention (VIP). These challenges include limited federal funding for this research, the lack of established or novel research methodologies, few collaborators with knowledge and/or experience in this field, and, most importantly, a small yet experienced number of established researchers who have been able to negotiate a successful research career in this field.

Because researching gender-based violence is challenging and requires great care to ensure safety for those participating in and conducting this research, having mentorship for those entering this field is crucial. Beginning researchers may be unaware or unready to deal with many of the issues that arise in this research area. Connections with advanced researchers could greatly mitigate these knowledge or experience gaps. Researchers and their teams may also experience safety risks in the forms of secondary trauma from hearing participant experiences to include anger, anxiety, and depression.

Beyond the academic pursuits of reading, writing and discussing VIP related issues, attributes of reflective practice include paying attention to emotions, triggers, and exploring healthy ways to deal with negative emotions (https://www.skillsyouneed.com/ps/reflective-practice.html). Mentorship groups are unique settings for work in handling secondary trauma reactions, and effective groups can provide ‘safe places’ to acknowledge, share and process these experiences with others. Participation in mentorship groups could thus result in less distress over time for new researchers.

Understanding gender-based violence and finding approaches to effectively intervene or prevent this violence is important given its magnitude, health impact and staggering economic impact at both personal and societal levels. Gender-based violence, also referred to as violence against women, has been defined to include intimate partner and sexual violence and harassment (Breiding et al. 2015). Worldwide, 30% (range 23.2%- 37.7%) of all women ever in a relationship have experienced physical and/or sexual violence by their intimate partner (Devries et al. 2013). While the personal cost of violence is borne by victims and cannot be comprehensively estimated, a recent global report has estimated the economic cost of physical IPV to be 2% of the global gross domestic product or 1.5 trillion US$ (http://www.unwomen.org/en/news/stories/2016/9/speech-by-lakshmi-puri-on-economic-costs-of-violence-against-women).

At the national level, the Centers for Disease Control and Prevention has played a pivotal role in supporting and funding this research to focus largely on the frequency and mental and physical health consequences of gender-based violence (Tjaden and Thoennes 2000; Smith et al. 2017; Basile et al., 2014 ). Recent lifetime estimates of sexual violence (SV) and intimate partner violence (IPV) reveal rates that were 2–3 fold higher among females (SV: 20% and IPV: 25%) than among males (7% and 10%, respectively) yet the health impact of these forms of violence were similar by sex (Smith et al. 2018). Recent estimates, based on NISVS data, indicate a cost of $122,461 per rape victim, which includes medical costs, lost productivity, criminal justice activities and other costs (Peterson et al. 2017). Similarly and based on NISVS data, the cost of IPV was estimated at $103,767 per female victim and $23,404 per male victim with a population economic burden of almost $3.6 trillion over victims’ lives (Peterson et al. 2018).

### Progress and Promise of Gender-Based Violence Intervention and Prevention Research

While conducting research in gender-based violence is difficult and rates of these forms of violence remain high, hope remains in the form of both intervention and prevention research findings. These promising findings provide justification for creating and supporting a national VIP mentoring program. While our field has progressed significantly in the measurement of violence and its personal and economic impact, the real challenge is that of finding new and effective approaches for violence intervention and prevention. The cost-benefit analyses conducted by Clark et al. (2002) is key to recognizing and enumerating the significant ‘pay off’ of federal funding to prevent violence. In their evaluation of the Violence Against Women Act of 1994 (VAWA-I; United States, 1994), these researchers found that this $1.6 billion investment in VAWA programs over 5-years saved $14.8 billion in net averted social costs measured as property losses, medical and mental health care costs, police response, victim services, lost productivity, reduced quality of life, and death (Clark et al. 2002). Fortunately, the Violence Against Women Act continues to be an important source of funding for intervention and prevention efforts particularly on college campuses.

The current CDC grant (U01 CE002668) is another example of a policy evaluation of the Violence Against Women Act and the implementation of the Campus Sexual Assault Elimination Act (SaVE) (Campus Sexual Assault Elimination Act Violence Against Women Reauthorization Act, 2013). With this funding, we worked with our CDC colleagues to evaluate the stipulation within the 2013 VAWA reauthorization that all institutions of higher learning (IHL) who received Title IX funding must offer bystander-based programming with the purpose of reducing risk of IPV and sexual violence on college campuses. In 2013, few bystander program evaluations existed to assist IHL in selecting bystander programming. Through CDC funding our team was able to recruit multiple colleges or universities, determine students’ exposure to bystander training programs, and assess the rate of sexual violence perpetration and victimization over 3–4 years (See Clear et al., 2019; Bush et al., 2020 and Davidov et al. 2020). Our purpose for this relative efficacy trial was to identify the components of bystander programs that had greater potential or direct impact on sexual violence over time. Thus, with Mentoring mcBEE all fellows participating in the mentoring program were also directly engaged in this policy evaluation.

Much remains to be accomplished in the upcoming decades by young researchers to meet the challenge of reducing gender-based violence and its associated personal and societal consequences. With rapid changes in electronic (and other) technologies, beginning researchers have skills to share with more senior researchers, such that they have much to contribute in both informing and expanding senior researcher perspectives and skills. Specific skills more experienced researchers can pass include elements of successful grant development and management, study design, recruitment, field methods, data analyses, and research communication. Thus, the mentoring model has creative and supportive benefits for both newer and more experienced researchers; their interactions benefit the field and ultimately have promise to reduce violence rates.

This publication contributes to the literature by providing a mentorship approach that will allow others to access the methods and instruments we used. This national mentorship approach can be used, adapted, and evaluated by others to test this strategy of supporting and mentoring junior faculty with interests within or outside VIP research.

### Description of Mentoring Multi-Colleges Bystander Efficacy Evaluation (mcBEE)

Mentoring mcBEE was created to operationalize the second aim for the CDC cooperative agreement (U01 CE002668) (Clear et al. 2019; Bush et al. 2020). This grant had two objectives. The first aim was highlighted above but both aims are outlined here: Aim 1) To grow communities of gender-based interpersonal Violence Intervention and Prevention (VIP) researchers within mcBEE by creating mentoring teams (hives) of senior and junior faculty; and Aim 2) to enhance the research trajectory of all members of this collaborative by increasing our research skillsets and exposure to newly-available technologies (e.g., techniques in surveying, statistical and cost analyses). Hereafter, this paper will focus on a description of the mentorship program developed to address Aim 2. Table 1 provides the timeline for the activities we highlight in the following section as we describe the creating of this mentoring program.

**Table 1:** Timeframes for Intervention Activities, Data Collection and Roles within Mentoring mcBEE

Activity	Time frame	Responsible lead
Aim. Build Gender-based VIP scholar-based research teams consisting of junior researchers and senior mentors	Fall 2015–2016	UK Data Coordinating Center (hereafter UK)
1. Finalizing agreements with our selected team of senior faculty as consultants (hereafter mentors) with excellent mentoring track records to serve as mentors for VIP fellows	Fall 2016	UK with VIP Mentors
2. Identifying junior faculty at recruited (or other) universities. Each must have a demonstrated interest or background in gender-based violence intervention or prevention (VIP) research. These faculty will serve as fellows in the Mentoring McBEE cohort across the 4 year cooperative agreement.	Fall 2016	UK with VIP Mentors and recruited McBEE universities
3. Foster communications within MmcBEE. Organize and host monthly calls/communication and host yearly research productivity retreats where project data are analyzed and manuscripts or grant applications are developed. Host site in Atlanta GA.	Monthly 2016–2019 Fall or Spring 2016–2019	UK with Mentors and Fellows
4. Prospectively measure research productivity and work/life satisfaction: Obtain CVs to determine baseline markers of research productivity and request researchers planning goals (including administrative and teaching obligations), measures of productivity beyond the CV (manuscript and grant submissions, presentations), research planning documents, and surveys of work life stress, satisfaction, control, and both physical and mental health and well-being.	Beginning of Spring 2016 term, then at the end of Spring and Fall terms for 2017, 2018 and 2019	UK with Mentors and Fellows

The Mentoring mcBEE program was modeled in part on the NIH funded ‘Building Interdisciplinary Research Careers in Women’s Health’ (BIRCWH) (Nagel et al. 2013). BIRCWH is a competitive training program for junior faculty with research interest in women’s health or sex differences research that provides funding to cover up to 75% of faculty time for research. With mentorship from senior faculty primarily affiliated at the university with the BIRCWH training award, junior faculty have up to three years to propose, conduct pilot research, and successfully compete for federal R or K equivalent funding. BIRCWH mentorship addresses 1) developing a productive scientific writing practice, 2) creating and maintaining a realistic manuscript development plan that fits with other negotiated academic duties, 3) plans and support for needed pilot research data, and 4) preparing a successful federal grant application.

#### Mentoring mcBEE Contrasts with BIRCWH in the Following Two Ways

First, Mentoring mcBEE sought to identify and support newer faculty across a national pool versus a single campus in receipt of BIRCWH funding. Given this national approach, Mentoring mcBEE used a ‘virtual’ mentorship network and relied on electronic communications via video or audio conferences, email and annual meetings. Secondly, in marked contrast with BIRCWH, Mentoring mcBEE did not have funding to support newer faculty to devote up to 75% covered time to their research for up to three years. The mentoring program we created as part of mcBEE was not part of an on-going national training program. The CDC funding, a cooperative agreement, is not a training grant. As investigators, we recognized the need for providing research mentorship and created the structure described here. We see the mentoring program as one example. We are collecting data to determine the effectiveness of this mentoring program to increase research productivity and improve career and life satisfaction. However, it is crucial that this (or any) mentoring program be funded such that each fellow’s time is covered, and therefore protected, to enhance their research productivity to develop and conduct VIP research.

### Creation of the Mentorship Network: mcBEE Hives

Our mentoring structure was based on identifying and linking advanced or senior VIP research faculty (hereafter, VIP mentors) with newer faculty who have a demonstrated commitment to VIP research (hereafter, VIP fellows). We sought to make these connections across universities, particularly where VIP mentorship was limited. Our primary goal was that of helping to grow the next generation of gender-based VIP researchers nationally.

#### We Began Recruitment by Identifying VIP Mentors

These mentors were senior faculty with proven performance in publications and funding in gender-based VIP research. The majority of mentors were invited and asked to provide a letter of support for the CDC grant. Two additional faculty were recruited as mentors given their roles as senior faculty at universities recruited to be part of the mcBEE study (Aim 1). These senior researchers served as very modestly paid consultants for their Mentoring mcBEE role. They provided advice and support to fellows and advice in fellows’ data collection, analyses and dissemination of findings. Because we used a BEE theme, we created a BEEhive such that each VIP mentor served as the ‘Queen BEE’.

Each fellow was recruited from campuses considering participation in mcBEE (CDC cooperative agreement Aim 1). Beyond our offer of collecting data at no cost to the university, the VIP mentorship program was a strong incentive for faculty engagement in mcBEE (Clear et al. 2019). Each recruited university was asked to nominate one faculty or staff with a strong research interest. We provided guidelines for the faculty we sought to support and strongly preferred nominations of those with a doctoral degree and a faculty position with a research expectation. This mentoring component was designed to enhance 1) research and work and life skills to be successful, productive and balanced faculty in academic settings and 2) networks with other (junior and more senior) researchers in the academic field of VIP research.

Fellows came from a range of research disciplines and were not necessarily in a faculty position at the time of nomination. Each fellow (or a local college designee) had a direct role in: 1) coordinating its university’s local college institutional review board (IRB) review and approval in coordination with UK research staff, 2) working with their campus registrar to obtain emails for undergraduate students ages 18–24 years of age, 3) coordinating survey launch, reminder emails, and delivery of incentives, 4) data cleaning, planning analyses, conducting and reviewing analyses (for each individual campus), and 5) communicating research findings to their college and to other audiences (publications, presentations).

Expectations of fellows included weekly calls with other ‘hive’ fellows and approximately monthly group calls with their hive mentor. Fellows were strongly encouraged to participate in individual mentor–fellow calls. Lastly, all mentors and fellows were strongly encouraged to attend annual mcBEE meetings. The costs of travel and lodging were covered each year with cooperative agreement funds.

With the recruitment of mentors and fellows, we were ready to create our mcBEE hives. Each hive had a ‘Queen BEE’ or mentor to two to four VIP fellows. Each of the seven hives was color-coded reflecting the seven mentors and each developed their own ‘personality’. An eighth hive included the principal investigators, CDC science officers, and research staff including the data manager / analysts and research coordinator (i.e., ‘Black Hive’ also referred to as the University of Kentucky (UK) Data Coordinating Center). Mentor/fellow hive ‘matches’ were made in March 2017 based on mutual requests of both mentors and fellows after our first annual ‘introductions’ meeting in November 2016. In this meeting, we requested that all participants provide a timed 2-min ‘Pecha Kucha’ presentation to introduce themselves to the group. These presentations represented the majority of this one-day meeting, held in association with a national meeting that many researchers were already attending.

A key element of Mentoring mcBEE was that of creating an infrastructure to stimulate new collaborations. Our annual in-person meetings were an important route to address this interaction. We planned and budgeted for annual meetings in 2016, 2017, 2018 and 2019. All Mentoring mcBEE fellows, mentors, staff and CDC scientists were encouraged to attend. The meetings have been held either at sites where other relevant conferences were held or in Atlanta, Georgia as a central location with close proximity to CDC scientists.

#### Mentorship Interactions and Resources

The purpose of the hives was that of building connections and a support network for fellows to make changes, if needed, to move forward toward increasing their research productivity and building work and life satisfaction. Weekly hive meetings were the principle means of interactions within hives. The day and time varied for each of the hives but the request was for a time that all in the hive could meet weekly. The goal was that fellows attend weekly hive meetings as frequently as possible. The expectation was for mentors to ‘meet’ with their hive on a monthly basis. Note that all activities within hives and between mentors and fellows were optional. The structure, resources, and activities were created as a set of offerings that, based on prior mentoring programs (https://www.facultydiversity.org), may work to improve academic productivity, job and life satisfaction, and continued interest in academic research. See the Appendix for descriptions of our hive toolkits (i.e., purpose of hives, how to create a writing practice, develop a research-prioritized planning schedule including weekly planning meetings with yourself).

The primary activity within the hives initially was developing or maintaining a daily writing practice for fellows. Fellows within a hive met weekly by conference call or Skype/Zoom. The purpose of weekly meetings was that of building connections with other fellows, providing support for other members and serving as accountability partners.

#### The Following Text Described Expectations of Mentors and Fellows

Expectations of mentors for consultancy contracts were: 1) a group electronic audio or video mentorship ‘call’ with hive fellows, 2) a call with mcBEE PIs to discuss needs, progress, or successes, and 3) one-on-one or group communications with fellows regarding research scheduling plans, publications and grant planning. The following text provides more detail on each of these mentoring activities.

- Monthly Hive Call. The purpose of this call was to check in with fellows as a group. We asked mentors to check-in with fellows to gauge their progress in prioritizing and completing their academic writing practice (at least 30 min per day), making and using semester and weekly research plans, determining whether selected common readings worked for the hive, and checking with fellows on their needs for manuscript or grant development support, other challenges, or mentorship needs. Hives structured this monthly call as it worked for the group. Most monthly mentoring calls ranged from 45 to 60 min.
- Quarterly Mentoring mcBEE check-in calls with the Black hive. These calls began as monthly and later became quarterly calls with all mentors. The purpose of calls were three-fold: 1) to report the challenges or needs within each hive’s members (particularly those needing additional support or resources), 2) to identify new grant or other research opportunities, research development or mentoring resources, or common readings that may be helpful within hives or for the annual meetings, and 3) to describe mentors’ challenges or needs to support mentorship or research trajectory (e.g. are there other preferred programs, other common readings, or other suggestions to improve the Mentoring mcBEE experience?). These calls ranged from 30 to 45 min.
- Individual communications with fellows. mcBEE principal investigators recommended at the beginning of the mentor–fellow communications that mentors review fellows’ Peak Performing Professor assessment (see below and Table 2) as fellows were willing to share these. These communications provided fellows the opportunity for individual time with their hive mentor to discuss issues that they may not be comfortable talking with others within their hive or within their campus.

**Table 2:** Planned Data Collection for cohort by time, and constructs measured

	Time frame of data collection (REDCap surveys)		
Constructs measured, source, internal consistency	Baseline Feb 2017	6 month follow-up: June 2017, 2018. Final: 2019	Annual Follow-up: Jan/Feb 2018, 2019
Current physical / mental health (and health relative to prior year) 17-item based on Medical Outcomes Study 36-item (Ware and Sherbourne 1992). Cronbach’s α = .52, Baseline	☑	☑	☑
Life Satisfaction. 3-items based on the Satisfaction with Life Scale (Pavot et al. 1991). Cronbach’s α = .84, Baseline.	☑	☑	☑
Optimism, trait anxiety, self-esteem. 7-items from the Life Orientation Test. (Carver et al. 2010). Cronbach’s α = .82, Baseline.	☑	☑	☑
Autonomy, participation, responsibility. 15 items based on Perception of Empowerment (Roller 1999) Cronbach’s α = .72, Baseline.	☑		
Work life balance. 2 general items, 12 items for past 6 months. Based on Daniels et al. 2000. Cronbach’s α = .83, Baseline	☑	☑	☑
Sage Senior Researcher Motivations measures how faculty prioritize their professional and mentoring choices. 18-items developed for mcBEE Cronbach’s α = .90, Baseline.	☑		☑
Assessment of faculty skills and orientation. Measures power (purpose), alignment, connection, energy, and professional roles and responsibilities in subscales. 30-items from Peak Performing Professor Assessment (Robison 2013) Cronbach’s α = 0.95, Baseline.	☑		☑
Size and support from Mentoring communities 30-items created for Mentoring mcBEE. Cronbach’s α = .84, Baseline.	☑	☑	☑
FINAL Survey only: Qualitative items including ‘Important phone call question’, Self & other fellow Mentoring mcBEE participation measure, and both quantitative & qualitative assessment of mentoring mcBEE experience. Created for mcBEE.		☑	

#### Example(s) of Mentoring mcBEE Hive Activities

The first hive assignment was a writing challenge. The goal of this challenge was that all mcBEE members commit to and conduct academic writing for at least 30 min per day. This approach was based on the Faculty Success Program’s Boot Camp (www.FacultyDiversity.org; Rockquemore 2019) because this program has been instrumental in helping young faculty develop the writing practices and other planning skills needed to become effective academics. Fellows were encouraged to meet weekly; mentors were encouraged to be on call on a monthly basis. Ultimately we hoped the hives would foster ‘protected time and space’ to work on joint manuscripts, grants, academic planning or career development decision-making venues.

To support developing a writing practice we identified materials, which addressed best or better practices to writing regularly and efficiently. We began our hive meetings using The Peak Performing Professor (Robison 2013) as a common reading in the summer–fall of 2017. The assignment was to work through this text provided to all as mentoring material. Baseline, follow up and the final mentoring surveys administered to fellows and mentors included the Peak Performing Professor (PPP, Robison 2013) assessment (see Table 2). This assessment was useful in determining how well individuals were doing regarding professional and work-life balance. The PPP assessment was broken into four peak performance areas (i.e., Power, Align, Connect, Energize) and then applied these practices to typical faculty roles and responsibilities at work and home. The Power subscale measured how finding and using your purpose motivates faculty (five questions). The Align subscale (five questions) measured how well faculty actions were aligned with required duties and with faculty power or purpose. The Connect subscale measured how faculty are connecting with helpful colleagues, friends and family for mental support (4 questions). The Energize subscale measured the extent to which faculty are actively sustaining themselves to handle the demands and stresses over time in an academic career (3 questions). The final subscale, Roles and Responsibilities, measured how well faculty are doing in their academic roles of teaching, research writing, service work, and in their personal roles (13 questions). Fellows and mentors had the choice to take the PPP assessment through individual survey invitations sent using Research Electronic Data Capture (REDCap) (REDCap; Harris et al. 2009). If they did participate in this mentoring evaluation, they received numeric feedback immediately after completing the PPP assessment. REDCap programmatically summed specific items and returned results in the form of a score sheet that broke down where participants were succeeding and where to focus attention. Fellows discussed their PPP assessment results with their hive with the purpose of determining how best to use their scores and the PPP text. While individual hives made their decisions regarding how to use the PPP text, our recommendation was to start work at the earliest assessment subscale with a focus on each individual’s lowest score where the lower score the area needing skill development. Using these scores, fellows could work through the book individually or in groups. The fellows’ scores on PPP self-assessment were also used as one tool to evaluate changes over time in productivity and life satisfaction (see Evaluation section below).

Opportunities for information and interactions, beyond weekly e-meetings, were established through our members-only website, monthly newsletters, occasional emails, and annual meetings. The following section describes these activities.

The Mentoring mcBEE private website was created and housed on a secure University of Kentucky server to help centralize documents; there was also a collection of resources such as funding opportunities, grant specifics, and upcoming conferences. We chose to make this website log-in secure in order to create a more private space to share Mentoring mcBEE project news and materials. Members had to create a username, then an administrator approved the website request granting access to Mentoring mcBEE participants.

This website served as a central location for project documents and materials. Resources such as grant applications faculty were willing to share, grant funding opportunities, and recent and relevant manuscripts were available to reference. The website included access to podcasts specific to Mentoring mcBEE, as well as recommended podcasts on various topics such as ‘How to Prioritize a Life that Matters’, ‘Dream Jobs’, ‘Do Less and Accomplish More’. Members could listen through Apple Podcasts, the Google Play store, or through SoundCloud by searching “mcBEE team.” There was a podcast introducing Mentoring mcBEE, a mock mentoring session podcast, and ten podcasts corresponding to each chapter summary of the text Peak Performing Professor presented by various women’s health or VIP researchers at the University of Kentucky.

Also included on the website were the original CDC cooperative agreement grant, annual meeting agendas (available on request from ann.coker@uky.edu), citation specifics to include on publication submissions, and mcBEE language for faculty CVs for mentors and fellows to reference. To support mcBEE grant efforts, recent (January 25, 2018) NIH changes were provided on the website in PowerPoint format in detail. Additional presentations on finding the right NIH funding mechanism as well as making sense of NIH R Grants were developed and provided with permission by the University of Kentucky Proposal Development Office.

A collaboration section on the website allowed any Mentoring mcBEE members to login and announce a position such as fellowship opportunities or scholar positions. This website was a place to share information and resources across our electronic network. For example, a ‘help wanted’ section facilitated cross-pollination of hives. If someone was writing a grant and was looking for a researcher with specific skill sets, they could turn to the group to explain the project and gauge interest in working together. A directory of mentors, fellows, CDC collaborators, and University of Kentucky contacts with pictures, title, affiliation and email addresses was provided on the website. Biographies of each member could be accessed by clicking on the picture or contact information. Under the virtual business card, research interests were listed and linked so that others with the same interest appeared in list form when selected.

In an effort to provide information to all Mentoring mcBEE members in a systematic way, newsletters were created. Using Mailchimp, we were able to incorporate and share upcoming tasks, milestones, grant information, conferences and deadlines, and information on the annual mentoring meeting. Newsletters sent monthly include creative displays of updates, introductions, important events to celebrate such as a new grant, published manuscript, promotion, or personal events such as a wedding or birth. Newsletters were used to advertise selected common readings and select our next reading by group vote for the next book to read and discuss.

Newsletters were also a good way to help coordinate Mentoring mcBEE member meet-ups at up-coming conferences. Our goal was that of helping connect fellows and mentors at common conferences. Through a calendar and a conferences networking survey, members could let the Black Hive know what conference(s) they would be attending by completing the global survey link. Before the conference, the Black Hive informed other Mentoring mcBEE members who else would be present at the same conference. The newsletter conference section also included a table of conference names, dates, location, and links to abstract calls and the conference websites so one could easily access conference information eliminating the need to search email inboxes.

#### Academic Planning Was another Skill Fostered within Hives

Specifically, this planning aimed at prioritizing research products such as manuscripts and future grants. Audio recordings of mentors’ experiences with developing academic plans were provided on our website as a resource. Examples of semester and annual planning documents, which describe research, teaching, service, and personal goals, were provided as excel worksheets. Time specific tasks outlined each goal. As a concrete example, if a grant submission was a desired research goal the time specific plan would include when each paper needed to support the grant submission would need to be developed, submitted, and published prior to the grant development and submission. The excel sheet included time for grant review and resubmission. Balancing grant submissions that have a clear due date with manuscript development, which typically does not have a due date, can be challenging. This challenge is more acute when teaching and service expectations all have time commitments with inflexible due dates. Building and maintaining scheduled time into an academic calendar is an essential skill for a successful researcher. See the Appendix for examples of how to use weekly planning meetings to support research productivity.

Within the hives, fellows and mentors provided input on academic research products to include manuscripts, grants, or other collaborative research products. In addition to these hive-based activities supporting research, we also offered support across hives termed ‘Cross Pollination.’ The goal of Cross-Pollination was that of facilitating networking across Mentoring mcBEE using the full network. As a ‘value add’ to this mentoring program we sought to provide 1) grant or manuscript review and 2) opportunities for networking and collaboration building from those outside the fellows’ hives (as needed or desired). To begin building the Cross-Pollination infrastructure, we first sought input from our mentors to determine who might be interested in providing reviews of research products. Next, we used Research Electronic Data Capture (REDCap; Harris et al. 2009), to launch a secure, web-based survey link for fellows to enter specifics on how we can help with both manuscripts and grant applications. This review process for many fellows was already occurring within their hives. The Cross-Pollination effort was created to expand these activities across hives and mentors. For manuscripts, fellows were asked to include the time frame and due date of the manuscript as part of their request as well as the specific areas they needed help in developing (e.g. research question, literature review, methodology, analysis plan, discussion), and targeted journals. The web-based survey provided a place for fellows to answer these questions, and to upload relevant documents such as a draft manuscript, abstract, or ideas. For grants, fellows were asked to provide the due dates, funding agency, funding announcement, and specific areas where they need help (e.g. analysis plan, collaborators, budget, sample size). Providing draft aims or research question(s) was encouraged. Requests were reviewed with engagement from other Mentoring mcBEE members with relevant expertise and feedback was provided back to fellows. A joint in-person grant workshop was conducted with junior faculty from mcBEE and BIRCWH at the University of Kentucky in October 2018 in conjunction with the Center for Research on Violence Against Women 2018 conference entitled ‘Campus responses to sexual misconduct: Navigating change at the Department of Education’.

#### Planned Evaluation of Mentoring mcBEE

Here we highlight the structure of our ongoing evaluation of Mentoring mcBEE. The following research question was posed and is being tested. ‘Does active participation in Mentoring mcBEE increase researchers’ (both mentors and fellows) network size and depth, research productivity, and academic skillset scores (from PPP assessment) across professional domains over time?’

#### Participants

All mentors and fellows, across all hives, were invited to participate in this prospective evaluation. See Table 2 for the timeline and data elements collected. The following were measures taken to ensure protection of human subjects for this cohort of researchers as participants. To evaluate the effectiveness of the mentoring program REDCap surveys were created and administered by the UK Center, who also sent automated electronic reminder invitations to complete launched surveys. Participation in this evaluation of the mentoring process was optional. Whether mentors or fellows decided to participate in the evaluation or not did not affect their participation in the mentoring and faculty skills development activities for this project.

Using REDCap for data collection ensured the safety and confidentiality of the transmission and storage of data, as REDCap operates behind a Cisco Systems firewall to protect data. Participants’ email addresses were used to send invitation links, and this data was retained to link individual responses over time through 2019. Contact information was stored in a separate survey instrument; therefore we could export survey data without identifiers. Final data sets including qualitative and quantitative data will be de-identified. To ensure transparency of the data analyses, neither principal investigator at UK, nor the data analyst from the UK Data Coordinating Center will be involved in the data analysis. Data analyses will be conducted by mcBEE affiliated faculty after the cohort data collection is complete (late Fall 2019). This research was reviewed and approved by the University of Kentucky, Office of Research Integrity, Medical Institutional Review Board (Protocol 15–0736-P2H).

Participation in this evaluation study involved completing short (15–20 min) electronic surveys at the beginning or end of each academic term (typically fall and spring) beginning in January 2017 through 2019. Answers may help researchers and administrators identify and disseminate ‘best practices’ for fostering academic faculty research productivity and work-life satisfaction.

Because we were using electronic surveys, a waiver of documentation of informed consent was requested and received. Participants were asked to read and electronically indicate consent. The consent form included options: 1) ‘Yes, I consent to participation in this study survey. My data will be used to evaluate the Mentoring mcBEE study. My answers to the PPP Assessment will be provided back to me as a tool to help me focus my attention on specific training needs’; 2) ‘I wish to complete only the PPP Assessment. My answer to the PPP Assessment will be provided back to me as a tool to help me focus my attention on areas of specific training needs’; 3) ‘No I do not wish to participate in the Mentoring mcBEE survey or complete the PPP Assessment’. Because the surveys included measures of general affect including depression, stress and anxiety, websites and toll-free phone numbers were provided to address related symptoms as well as substance use disorders at the end of all surveys.

The baseline and the final mentoring mcBEE surveys included a shortened version (seven of ten items) of the Life Orientation Test developed to assess individual differences in generalized optimism versus pessimism (Carver et al. 2010), a 3-item measure of general life satisfaction based on the Satisfaction with Life Scale (Pavot et al. 1991), and a reduced 12-item measure of work life balance based on the Work Life Balance Manual (Daniels et al. 2000). Two additional measures were developed for this evaluation. A 30-item measure of the size and support gained from mentoring networks within and outside academic settings. The second measure, developed specifically for evaluating the impact of MmcBEE for mentors, was the 18-item Sage Senior Researcher Motivations Measure. In Table 2, the psychometric properties for these quantitative measures are provided.

The final survey asked questions specific to participation in Mentoring mcBEE. We sought feedback from fellows and mentors alike regarding whether they would recommend this mentoring and work-life balance program to other faculty with similar research interests. Additional questions assessed agreement with the following questions: ‘I benefited from participating in mcBEE’, ‘My planning skills improved through participating in mcBEE’, ‘My stress levels declined through participating in mcBEE’, ‘My research productivity increased through participating in mcBEE’, ‘My personal life has improved as a result of participating in mcBEE’, ‘My research network has increased as a result of participating in mcBEE’. Open-ended notes boxes gave participants the opportunity to provide feedback on what they see as the strengths of the program, the weaknesses of the program, what aspects could/should be eliminated, and what training, skill building, resources or networking could/should be added. The longitudinal surveys helped to quantify time spent on research community engagement, and a qualitative assessment of satisfaction with the collaborative research community. Also individualized data collection measuring self-perceived research skills, strengths, weaknesses, mental and physical health and well-being, and work and life satisfaction and perceived balance.

#### Evaluation Plan

We hypothesized that greater participation with the mcBEE mentoring activities would, over time, increase academic productivity and improve work and life satisfaction. A prospective cohort design was planned for this evaluation measuring academic research skills acquired, goals achieved, and research productivity for both fellows and mentors. The primary exposure for this cohort was the degree of involvement in the mentoring activities. Involvement was defined as participation in weekly hive e-meetings with other fellows, monthly e-meetings with the hive mentor, attendance at annual Mentoring mcBEE national meetings, and use of grant or manuscript development resources through Mentoring mcBEE. Participation was measured over time throughout the cooperative agreement timeframe. Primary indicators of research productivity included the number of grants submitted or were funded, and the number of manuscripts under review or were published. Other indicators of research or academic success included promotion to the rank of associate or full professor, other desired position changes, receipt of teaching, research or service awards, and desired changes in family life such as births or marriages. Data collection is ongoing through late 2019. Analyses will be conducted among all participants and separately among mentors and fellows. See Table 2 for time frames for launching the REDCap surveys and survey content.

#### Analysis Plan

The ultimate analytic question was to what degree did Mentoring mcBEE participation enhance the research trajectory of all members of this collaborative. Both quantitative and qualitative methods will be used to analyze de-identified data. This evaluation uses a prospective cohort design. Fellows’ current biosketches and/or CVs, as well as REDCap survey data will be used to conduct pre–post qualitative assessment of the mentoring relationship, scholar productivity and work / life satisfaction. Data items include socio-demographic characteristics, academic backgrounds, areas of expertise, faculty rank, self-assessments, and productivity. Qualitative measures of Mentoring mcBEE’s impact for fellows will measure their perception of this mentoring program’s value toward achieving career accomplishments such as whether (1) they became mentors to others, (2) remained in gender-based VIP research, and (3) would recommend this mentoring initiative to others.

More quantitative outcome measures will include the following metrics: number of manuscripts developed, submitted, and published by fellows, with or without a gender-based VIP focus; number of grants submitted and granted by fellows with and without a VIP focus; number of presentations communicated to national or international settings by fellows with and without a VIP focus; and the number of fellows promoted or reaching other self-identified success milestones.

Qualitative assessments of the fellow-mentor relationship, changes in scholarly productivity, and work/life satisfaction will be carried out via document analysis and through content analysis of answers to open-ended questions on REDCap surveys. Specifically, individual fellow’s CVs and biosketches will be examined to capture significant changes in faculty rank (e.g., promotion to Associate Professor) and the number of presentations and publications with and without a VIP focus before and after participation in Mentoring mcBee. Fellows will also report this information in REDCap surveys and both sources will be compared to enhance validity. Open-ended survey responses on REDCap surveys about fellows’ perceptions about the value of the Mentoring mcBee program and changes in work/life satisfaction will be analyzed via content analysis with open coding. Each response will be open coded and clusters of content will be grouped together into thematic categories for each question.

The unit of analysis for this cohort will be the individual participant (fellow or mentor) followed over time. The primary analysis will be to estimate changes in outcomes from pre-mentoring to end of study; estimates will be provided with 95% confidence intervals. The primary exposure or independent variable will be the a) degree of exposure to Mentoring mcBEE (intensity) measured by self-report and estimations from other hive members and b) the duration (person months) of engagement within Mentoring mcBEE. Exposure measures will be treated as both continuous and categorical variables. The primary outcomes of interest are the size and depth of fellows (or mentors) research network and collaborations, 2) their research productivity, and 3) their PPP scores in total and by subscales. Size and depth of one’s research network were measured by the number (size) of others who support, collaborate, or advise the fellow within or outside the fellow’s institution. Depth was measured via a qualitative assessment of the degree to which others provide tangible support and listen to the fellow, offer new approaches, actively participate in research writing or conduct, and provide direction or advice if requested.

Scores for outcomes will be calculated and summarized as continuous variables (n, mean, SD, median, range) by time point; change and percent change scores will be calculated from pre-mentoring assessment and summarized as continuous variables. Profile plots describing individual-level trend lines over time will be used to visually describe participants overall and by hive. While the primary unit of analysis is the individual, scores will also be aggregated into cluster means for by hive analyses. Estimated change scores will be compared by exposure levels using linear regression models with pre-mentoring values included for adjusted estimates. Demographic attributes, roles (mentor or fellow), and rank will also be explored as confounders and potentially modifiers of the association between Mentoring mcBEE exposure and hypothesized increases in outcomes (i.e., PPP scores, research productivity, and work / life satisfaction). To explore trajectories over time, linear mixed models will be used to account for repeated measures and potential changes over time. Time will be included as an interaction term with Mentoring mcBEE exposure. As an additional exploratory analysis, multivariate methods such as latent class analysis and classification and regression trees may be used to identify features of successful mentoring programming.

The current paper provided a review of a mentoring program for junior faculty engaged in gender-based interpersonal violence intervention or prevention research. This review contributes to the existing literature by providing a ‘toolkit’ of the recruitment process, mentoring delivery, and prospective cohort evaluation plan with specific survey descriptions. The methods for providing group and individualized mentorship for recruited faculty across multiple colleges were provided with the express purpose of inviting other to use, adapt, and evaluate this approach in the near future. The described upcoming evaluation of Mentoring mcBEE will provide qualitative and quantitative evidence regarding the efficacy of the outline program to meet our stated goals.

As investigators, we recognized the need for providing research mentorship and created the structure described here. We see the mentoring program as one example. We are collecting data to determine the effectiveness of this mentoring program to increase research productivity and improve career and life satisfaction. However, it is crucial that this (or any) mentoring program be funded, such that each fellow’s time is covered, and therefore protected, to enhance their research productivity to develop and conduct VIP research. To grow the next generation of researchers in the field of gender-based violence intervention and prevention we need to additionally foster and recruit young undergraduates into this field. The federally-funded STEM program, which engages female and male high school and college students in seeking careers in science, technology, engineering and mathematics, may be a good model to increase the awareness and legitimacy of research and practice-based careers in gender-based violence intervention and prevention (VIP).

Gender-based violence continues to disproportionately impact the lives of women, girls, and sexual minorities regardless of biological sex. Facing this fact by ensuring the educational and financial support needed to create and maintain the workforce who can address this health threat is the right thing to do, and now is the time to make a ‘future without violence’ a reality.

## Appendix

1. Purpose of Small Hives = Support YOUR Research Productivity

How? Increase the time you have to WRITE

One of the biggest barriers researchers face in meeting their personal and professional goals is finding time for writing. But writing is the most essential activity for researchers. Writing can take many forms from reading scientific literature, creating an outline for an abstract, crafting a section for a manuscript, drafting an abstract for a conference, or working on a proposal for a book. Publications are our data to support our annual reviews, salary increases, promotion and tenure decisions. Our publication history also fuels our grant funding potential.

Typically faculty have difficulty finding time to write because other people or issues are more pressing. Any task with a due date appears more important that a manuscript because these usually do not have a due date. You need to create your own due dates for your essential research products.

With mcBEE small hives, we launched our writing challenge. Our approach is based on the Boot Camp from the Faculty Success Program developed by Kerry Ann Rockquemore of the National Center for Faculty Development & Diversity (https://www.facultydiversity.org/home). The Boot Camp was designed for advanced graduate students, post-docs, tenure-track faculty, AND tenured faculty who seek to increase their research productivity through accountability and peer support. The goal is not only to increase productivity but improve faculty work and life balance and happiness. In contrast with the Faculty Success Program 12-week boot-camp, mcBEE hives continued to meet weekly throughout this CDC funded grant.

For weekly hives meetings, fellows worked on developing achievable professional goals and realistic plans to meet goals, developing and maintaining a writing practice, observing and learning from writing or planning challenges, and creating supportive hives that processes day-to-day challenges, holds members accountable as needed, and celebrates members’ successes.

mcBEE hive support accountability calls were an essential part of the MmcBee program. Monthly calls with mentors and weekly calls with other fellows provided a consistent mechanism for junior faculty to receive support and accountability with the ultimate goal of developing a daily writing routine of at least 30 min a day. By learning to prioritize research and writing, fellows will cultivate new habits that will enable them to meet their short- and long-term academic and personal goals. We began the weekly calls with a focus on creating and maintaining the writing practice. Over time hives became gestational units within which fellows developed joint manuscripts, book chapters and grant applications.

**Table:**

Example. Manuscript list and status			
Papers in development, progress, in press	Target Journal 1st, 2nd, 3rd choices	Tasks and Dates	Status (Person Responsible for next step)
Brief description (not necessarily the title) within each of the above 3 groups			

### Examples of annual, quarterly, or weekly **research** planning schedules

**Table:**

Spring 20xx Example	
Fellow Name	Last Updated: Date
Personal Goals (example goals with time frames if possible)	
1. Exercise.	
2. Time with partner, friends, family.	
3. Take one full weekend day/night off per week.	
Research Goals (example)	
1. Submit one R/K grant.	
2. Submit 4 research manuscripts.	
3. Publish 2 first authored manuscripts.	
4. Attend and present at one national meeting.	

Include goals within other work/life domains

*Monthly versus quarterly time intervals may be more useful but the point is to keep this bigger picture planning document realistic and management. This timeline should neither be overwhelming nor detailed. The time frames for grants will not likely be the same for manuscripts

**Many find it useful to color code different activities on a planning document

### Moving from semester to weekly planning meetings

Having the semester or academic year planning completed is a great start. You will know the bigger picture goals like submitting those papers to support your more competitive grant, submission.

Some faculty find that a planning meeting with yourself at the beginning or end of each workweek is helpful to stay on track and get those goals you set for yourself met.

We provided examples of quarterly and weekly planning ‘calendars’ in the prior pages.

Here we talk through what you might do on a week-to-week basis to 1) plan your upcoming week and 2) evaluate how well you did in getting planned tasks accomplished. If you are anything like me, I am far too optimistic and over plan what I can accomplish. I also do not plan ‘celebrations’ when things are completed. A celebration for me might be going home, taking a walk, or a tea treat. Building in celebrations of targeted accomplishments are an affirmation of the planning process.

Some find that a weekly planning meeting works best on Friday afternoon. Others find that Sunday night or Monday morning is a good time to schedule your activities. Monday morning works well for me. I also *try* to include time-framed activities on my electronic calendar so that I have personal accountability. Honestly, things move into the next week but they do get done if they are on my calendar. The goal of this meeting with yourself is to be ready for the week ahead knowing what you want and need to work on to get the research writing goals accomplished. This meeting should not take more than 30 min. Using a timer will help you keep the meeting short and productive.

To get started on your weekly plan you will need

- your quarterly and weekly plan
- your calendar (electronic or ‘hard copy’
- Any ‘to do’ lists you may have made throughout the week

1. If you have not already done so block off prior commitments such as faculty meetings, course prep and teaching class, or other training or national meetings you may have scheduled.
2. Create a list of tasks that need to be completed on a regular basis and time the time these should take. If these are occurring, include these on your calendar.
3. Next, introduce new tasks linked to your quarterly or weekly calendar. You might use a brain dump or dream book approach to list things you need to do or really want to do.
4. Prioritize the list to start with activities that directly relate to your tenure and promotion, meaning getting papers published and grants submitted. (See Covey’s Quadrants as another tool to help with prioritizing)

Here is a beginning of a to do list as an example.

**Table:**

Weekly To Do List	
TASK	FREQUENCY OR DUE DATE
Research/Writing	
Begin writing introduction section for xx Paper	30 min Mon, Wed, Fri

The Covey Quadrants are a useful tool to create your weekly plan thus operationalizing your semester plan. You want to stay in the top two quadrants if at all possible. Prioritize what is important to you and YOUR success as a faculty member.

**Table:**

Covey Quadrants *	
1. Urgent and Important	2. Important and not urgent
3. Urgent and not important	4. not urgent and not important

*Covey, Stephen R. The **7 Habits of Highly Effective People**: Restoring the Character Ethic. New York: Free Press, 2004
